# Multimodal imaging of Hurler syndrome-related keratopathy treated with deep anterior lamellar keratoplasty

**DOI:** 10.1186/s12886-020-01689-2

**Published:** 2020-10-31

**Authors:** Elodie Da Cunha, Cristina Georgeon, Nacim Bouheraoua, Marc Putterman, Françoise Brignole-Baudouin, Vincent M. Borderie

**Affiliations:** 1 GRC32, Transplantation et Thérapies Innovantes de la Cornée, Sorbonne université, Centre Hospitalier National d’Ophtalmologie des 15-20, 28 rue de Charenton, 75571 Cedex 12 Paris, France; 2 Laboratoire (2), Centre Hospitalier National d’Ophtalmologie des 15-20, Paris, France

**Keywords:** Case report, Deep anterior lamellar keratoplasty, Hurler syndrome, In vivo confocal microscopy, Optical coherence tomography

## Abstract

**Background:**

Hurler syndrome-associated keratopathy is an exceedingly rare corneal disorder that requires corneal transplantation in advanced stages. Precise assessment of the corneal condition is necessary for deciding which type of keratoplasty (i.e., deep anterior lamellar or penetrating) should be proposed. We aimed to confront the results of multimodal imaging with those of histology in a case of Hurler syndrome-associated keratopathy.

**Case presentation:**

A 16-year-old patient with Hurler’s syndrome treated with hematopoietic stem cell transplantation was referred for decreased vision related to advanced keratopathy. The patient was treated with deep anterior lamellar keratoplasty (DALK) in both eyes with uncomplicated outcome. Visual acuity improved from 0.1 (20/200) preoperatively to 0.32 (20/63) and 0.63 (20/32) after transplantation. The corneal endothelial cell density was 2400 cells/mm^2^ in both eyes 3 years after transplantation. In vivo confocal microscopy (IVCM) and spectral domain optical coherence tomography (SD-OCT) were performed preoperatively. The corneal buttons retrieved during keratoplasty were processed for histology. In SD-OCT scans, corneal opacities appeared as diffuse stromal hyperreflectivity associated with increased corneal thickness. IVCM showed diffuse cytoplasmic granular hyperreflectivity and rounded/ellipsoid aspects of keratocytes, presence of small intracellular vacuoles, and hyperreflective epithelial intercellular spaces. Bowman’s layer was thin and irregular. The corneal endothelium was poorly visualized but no endothelial damage was observed. Histology showed irregular orientation and organization of stromal lamellae, with the presence of macrophages whose cytoplasm appeared clear and granular. A perinuclear clear halo was visible within the epithelial basal cells. Bowman’s layer featured breaks and irregularities.

**Conclusions:**

The observed corneal multimodal imaging features in mucopolysaccharidosis-related keratopathy were concordant with histology. Compared with standard histology, multimodal imaging allowed additional keratocyte features to be observed. It revealed both morphological and structural changes of all corneal layers but the endothelium. This information is essential for therapeutic management which should include DALK as the first-choice treatment in case of impaired visual acuity.

## Background

Mucopolysaccharidosis type I (MPS I) is a very rare autosomal recessive disorder affecting lysosomal metabolism [1], with an overall prevalence of 1/100000 [2]. Characterized by mutations of a ubiquitous lysosomal enzyme, α-L-iduronidase (IDUA), which is involved in glycosaminoglycan metabolism, it leads to intra- and extracellular accumulation of dermatan and heparan sulfate which results in cell, tissue, and organ damage and dysfunction. MPS I is subdivided into 3 syndromes. Hurler syndrome (MPS IH) is the most severe form with multi-organ and progressive neurological involvement. The large clinical heterogeneity of mucopolysaccharidoses is partly explained by the large number of existing mutations, some of which permit some residual enzymatic activity [3].

The main clinical manifestations include dysmorphism, hydrocephalia, mental retardation, hepatomegaly, skeletal, cardiac, and pulmonary malformations. The main ophthalmological disorders are corneal opacities, glaucoma, retinitis pigmentosa, papillary edema, and optic nerve atrophy.

Hurler syndrome-associated keratopathy leads to impaired visual acuity in advanced stages requiring corneal transplantation to restore vision. Penetrating keratoplasty (PK) and deep anterior lamellar keratoplasty (DALK) can be proposed with known advantages associated with the latter technique, such as lower rejection risk and higher survival of the corneal endothelium. Precise assessment of the corneal condition is necessary for deciding which type of keratoplasty should be proposed. Successful DALK surgery in MPS I has been reported in 4 patients, whereas reported data concerning PK in MPS I is only poor with graft failure observed in 2 out of 3 patients [4–8].

Corneal multimodal imaging, combining in vivo confocal microscopy (IVCM) and spectral domain optical coherence tomography (SD-OCT), is an essential tool for the diagnosis and characterization of many ocular surface diseases [9, 10]. To our knowledge this approach has not been reported yet to assess Hurler syndrome-associated keratopathy. We report multimodal imaging and histology of the cornea in a patient with Hurler syndrome who underwent deep anterior lamellar keratoplasty (DALK). 

## Case presentation

**Fig. 1:**
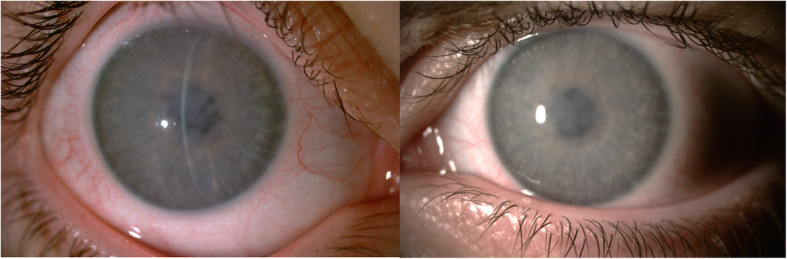
Hurler syndrome-associated keratopathy observed with slit-lamp

A 16-year-old female patient with Hurler’s syndrome treated with hematopoietic stem cell transplantation was referred for decreased vision. The diagnosis had been made by quantification of α-l-iduronidase and gene sequencing. Systemic disorders in this pediatric patient included dysostosis, arachnoid cysts and sleep apnea. Corrected visual acuity was 0.1 (20/200) in both eyes. Slit lamp examination revealed diffuse, whitish, milky, full-thickness stromal opacities (Fig. 1). Intraocular pressure was 28 mmHg in both eyes. Corneal hysteresis was increased (Corneal Hysteresis, 18.6/20.9 mmHg; Corneal Resistance Factor, 20.7/21.5 mmHg) and corrected intraocular pressure (IOPcc) provided by the ORA device (Reichert, Inc., Depew, NY) was normal (17/11 mmHg). Fundus was unremarkable and electrophysiological examinations were within normal limits (ERG, response to the lower limit of normal with no overall deficit; PEV, no major conduction disorders in the visual pathways). No history or evidence of optic nerve involvement was found in this patient.

A DALK was performed in the left eye and, 3 years later, in the right eye. A type 1 big bubble was obtained after air injection in the posterior stroma [11]. The posterior bed consisted of the predescemetic Dua’s layer in the 6-mm central zone and the posterior stroma in the 6–8.25 mm peripheral zone. An 8.25-mm graft was sutured after Descemet’s membrane removal using a combination of 8 interrupted sutures and 16-bit running suture. Long-term follow-up was unremarkable, i.e., clear grafts, improved vision, normal intraocular pressure, no rejection episodes. Four years after left eye transplantation, visual acuity was 0.32 (20/63, RE) and 0.63 (20/32, LE). Central corneal thickness (CCT) was normal (RE, 530 μm; LE, 520 μm). The corneal endothelial cell density was 2400 cells/mm^2^ in both eyes. The patient was satisfied with the results of transplantation which provided her with improved quality of life (Table 1).

**Table 1:** Shows a timeline of the episode of care

Time point (year)	Patient age (years)	Intervention	Workup
0	16	DALK, LE	Visual acuity, slit-lamp, SD-OCT, IVCM
1	17		Visual acuity, slit-lamp, SD-OCT, SM
2	18		Visual acuity, slit-lamp, SD-OCT, SM
3	19	DALK, RE	Visual acuity, slit-lamp, SD-OCT, SM
4	20		Visual acuity, slit-lamp, SD-OCT, SM

*DALK* deep anterior lamellar keratoplasty, *IVCM* in vivo confocal microscopy, *LE* left eye, *RE* right eye, *SD-OCT* spectral domain optical coherence tomography, *SM* specular microscopy

Preoperatively corneas were assessed with SD-OCT (RTVue-100©, Optovue Inc., Fremont, CA) and In vivo confocal microscopy (Heidelberg Retina Tomograph II/Rostock Cornea Module, Heidelberg Engineering GmbH, Heidelberg, Germany). Corneal buttons obtained during keratoplasty were processed for histology and stained with hematoxylin-eosin-safran, Schiff periodic acid, and Masson’s trichrome.

**Fig. 2:**
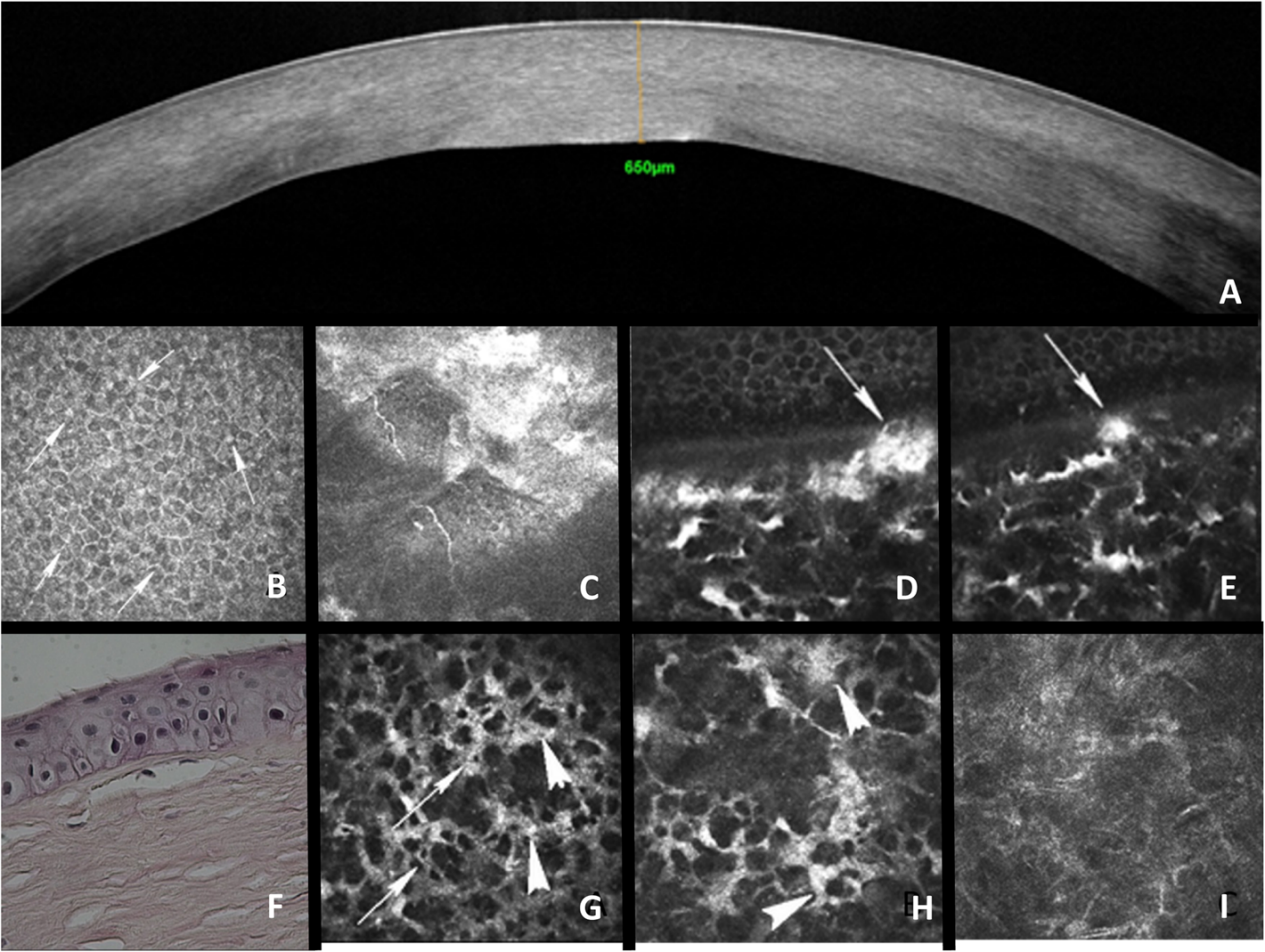
Multimodal imaging and histology of Hurler syndrome-associated keratopathy. Both extracellular (arrows) and intracellular (arrowheads) deposits are observed. **a** SD-OCT cross-section. **b-e** IVCM of the corneal epithelium and anterior stroma. **f** histology. **g-i** IVCM of the corneal stroma

SD-OCT showed diffuse hyperreflectivity with normal endothelial reflectivity. CCT was 695 μm in the right eye and 662 μm in the left eye (Fig. 2a).

In Vivo Confocal Microscopy was performed by a trained senior orthoptist. It showed changes in corneal layers including diffuse hyperreflectivity of the corneal epithelium, presence of granulations within the epithelial intercellular spaces, sub-basal nerve plexus irregularity and rarefaction, hyperreflective keratocytes featuring morphological changes (i.e., round or ellipsoid shape, activated keratocytes, granular cytoplasm, presence of small dark intracellular images forming intracytoplasmic vacuoles) (Fig. 2b, c, g, h, i). Vacuoles were located close to the keratocyte nuclei. Hyperreflective deposits were observed in the anterior stroma associated with changes in Bowman’s layer, which appeared irregular and thinner (Fig. 2d-e). Stromal opacification resulted in poor endothelial cell visualization. No damage to the corneal endothelium was found.

Histology (Fig. 2f) showed no differences between fellow corneas. The superficial epithelial cells appeared normal. Basal epithelial cells were distributed in different planes. They featured a perinuclear clear halo. Bowman’s layer was irregular with variable thickness and presence of isolated keratocytes just below Bowman’s layer. Stromal changes were mainly located in its anterior part, including irregular lamellae, presence of clear cytoplasmic mononuclear macrophage cells, and PAS-positive granulations.

## Conclusions

In our patient, SD-OCT showed diffuse hyperreflectivity, uniformly distributed across the entire corneal thickness and associated with increased CCT. Ahmed et al. [12] found OCT to be useful in mucopolysaccharidosis for assessing CCT and anatomy of the iridocorneal angle. Age-dependent increase in CCT was reported in all mucopolysaccharidosis. Other studies did not find this correlation [13].

We observed changes affecting all layers in In vivo confocal microscopy, with diffuse hyperreflectivity and granular hyperreflectivity between epithelial cells. The latter has been previously reported in MPS IS and MPS I-HS [14, 15], demonstrating epithelial involvement in MPSs. In histopathology we observed alterations in the basal epithelial layer with perinuclear halo as reported by others in MPS I-S [16] and MPS I-HS [15]. Conversely, Huang et al. [17] reported unremarkable corneal epithelium. Keratocyte changes included rounded ellipsoid shape, hyperreflective cytoplasm, and intracytoplasmic vacuoles [14, 15] in accordance with histology, which demonstrates intra- and extracellular accumulation of glycosaminoglycans [17]. Stromal deposits are associated with increased variability of both fibril diameter and inter-fibril distance and the presence of long-spacing collagen fibrils that contribute to stromal opacification [17, 18].

Contribution of In vivo confocal microscopy to corneal endothelium assessment before keratoplasty is limited by poor visualization of endothelial cells. No damage to the corneal endothelium was found in our patient. Nevertheless, SD-OCT showed normal endothelial reflectivity. Endothelial involvement in MPS is controversial and probably depends on the disease stage. In histology, endothelial intracellular vacuoles are not constant [15–17]. Our patient featured unremarkable preoperative endothelial assessment, both with IVCM and SD-OCT. Post-operative specular microscopy confirmed the absence of endothelial damage and normal endothelial function.

Enzyme replacement therapy and hematopoietic stem cell transplantation are the major treatments of mucopolysaccharidoses [19]. However, their effect on corneal opacification remains controversial [20–22]. In addition, retinal involvement may progress with time after hematopoietic stem cell transplantation [22]. Keratoplasty is the reference treatment of severe corneal opacification, allowing visual rehabilitation if retinal function is maintained. Favorable results were reported after penetrating keratoplasty [23]. However, as MPSs mainly involve epithelium and stroma, DALK seems more suitable in young patients [24] because it reduces the risk of endothelial rejection. Few DALK cases (8 eyes from 4 patients) have been reported in mucopolysaccharidosis patients [4–6]. Our patient presented good visual recovery, uneventful outcome, and absence of corneal opacity recurrence. Similar results were reported in a series of 4 eyes from 2 patients with Hurler’s syndrome and Hurler-Scheie syndrome and in a patient with Hurler-Scheie syndrome undergoing concurrent enzyme replacement therapy [5, 6].

Multimodal imaging of mucopolysaccharidosis-related keratopathy revealed both morphological and structural changes of all corneal layers but the endothelium. This information is relevant for therapeutic management which should include DALK as the first-choice treatment in case of impaired visual acuity.

## Data Availability

Anonymized data used to support the findings of this study are available from the corresponding author upon request.

## Abbreviations

DALK: Deep anterior lamellar keratoplasty
IVCM: In vivo confocal microscopy
LE: Left eye
MPS: Mucopolysaccharidosis
RE: Right eye
SD-OCT: Spectral domain optical coherence tomography
SM: Specular microscopy
